# HR-NeRF: advancing realism and accuracy in highlight scene representation

**DOI:** 10.3389/fnbot.2025.1558948

**Published:** 2025-04-16

**Authors:** Shufan Dai, Shanqin Wang

**Affiliations:** Chuzhou Polytechnic, Chuzhou, China

**Keywords:** scene representation, view synthesis, image-based rendering, volume rendering, 3D deep learning, spectral bias

## Abstract

NeRF and its variants excel in novel view synthesis but struggle with scenes featuring specular highlights. To address this limitation, we introduce the Highlight Recovery Network (HRNet), a new architecture that enhances NeRF's ability to capture specular scenes. HRNet incorporates Swish activation functions, affine transformations, multilayer perceptrons (MLPs), and residual blocks, which collectively enable smooth non-linear transformations, adaptive feature scaling, and hierarchical feature extraction. The residual connections help mitigate the vanishing gradient problem, ensuring stable training. Despite the simplicity of HRNet's components, it achieves impressive results in recovering specular highlights. Additionally, a density voxel grid enhances model efficiency. Evaluations on four inward-facing benchmarks demonstrate that our approach outperforms NeRF and its variants, achieving a 3–5 dB PSNR improvement on each dataset while accurately capturing scene details. Furthermore, our method effectively preserves image details without requiring positional encoding, rendering a single scene in ~18 min on an NVIDIA RTX 3090 Ti GPU.

## 1 Introduction

Novel view synthesis has been a persistent challenge in computer vision and graphics. Utilizing deep learning to interpret 3D scenes from sparse image sets has wide-ranging applications in entertainment, virtual and augmented reality, and other fields. Emerging neural rendering techniques have recently enabled photorealistic image quality for these tasks. One of the most prominent recent advances in neural rendering is NeRF (Mildenhall et al., 2020) which, given a handful of images of a static scene, learns an implicit volumetric representation of the scene that can be rendered from novel viewpoints. Although the current neural rendering technology has achieved leading image rendering quality, it still does not perform well in terms of model acceleration and image specular reflections detail. By sampling the 3D coordinates in the scene, and using the MLP to infer the density of the location and the view-dependent color value, NeRF renders compelling photorealistic images of 3D scenes from novel viewpoints using a neural volumetric scene representation. Volumetric neural rendering incurs a significant computational burden due to stringent sampling requirements and the high cost of neural network queries, leading to substantially prolonged processing times. To address this limitation, we adopt the dense voxel grid linear interpolation strategy proposed by Sun et al. (2022) to generate the scene's density and view-dependent color features.

**Figure 1:**
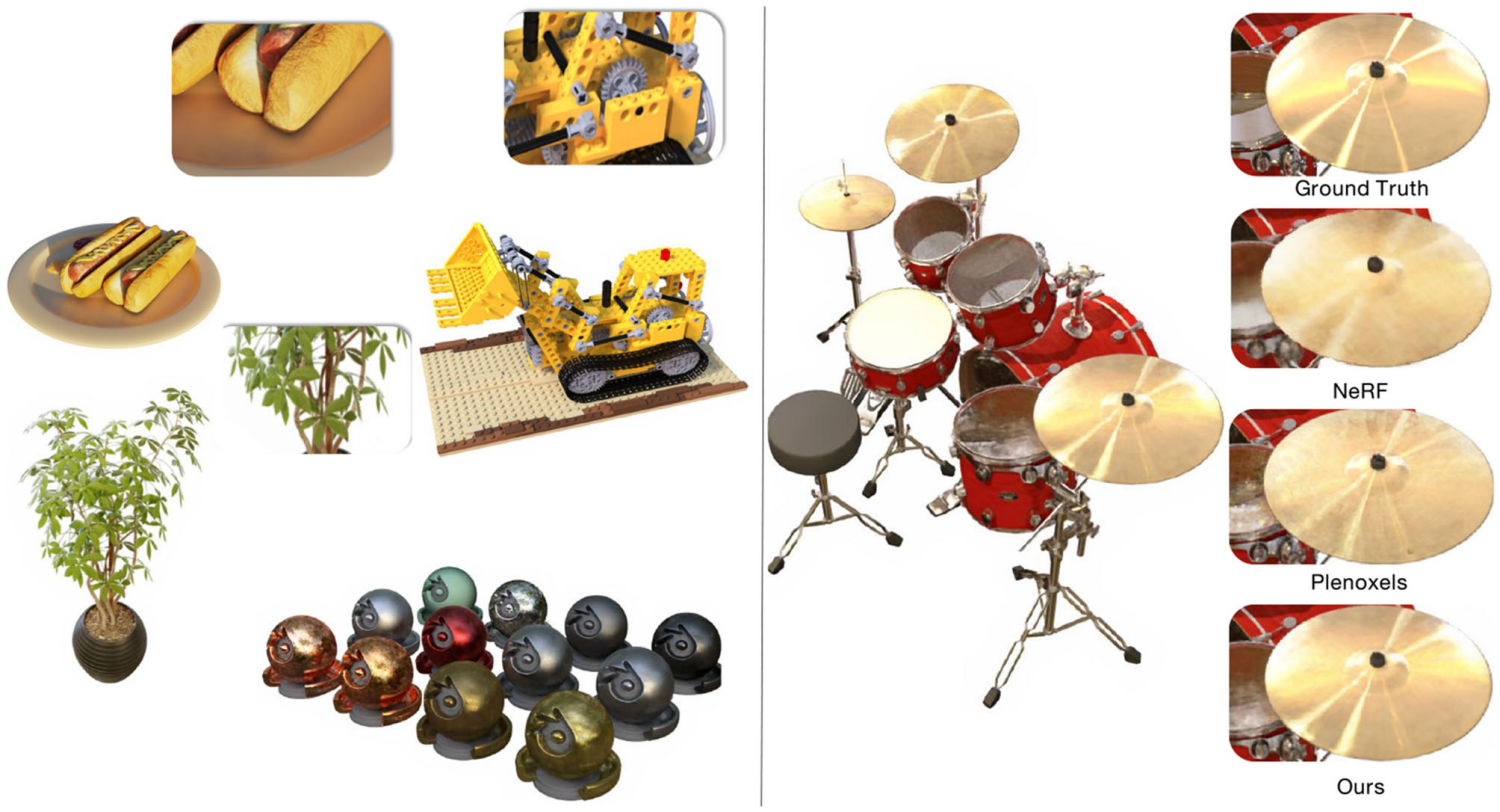
We present a method to represent complex signals such as specular reflections. Our method is able to match the expressiveness of coordinate-based MLPs while retaining reconstruction and rendering speed of voxel grids.

**Figure 2:**
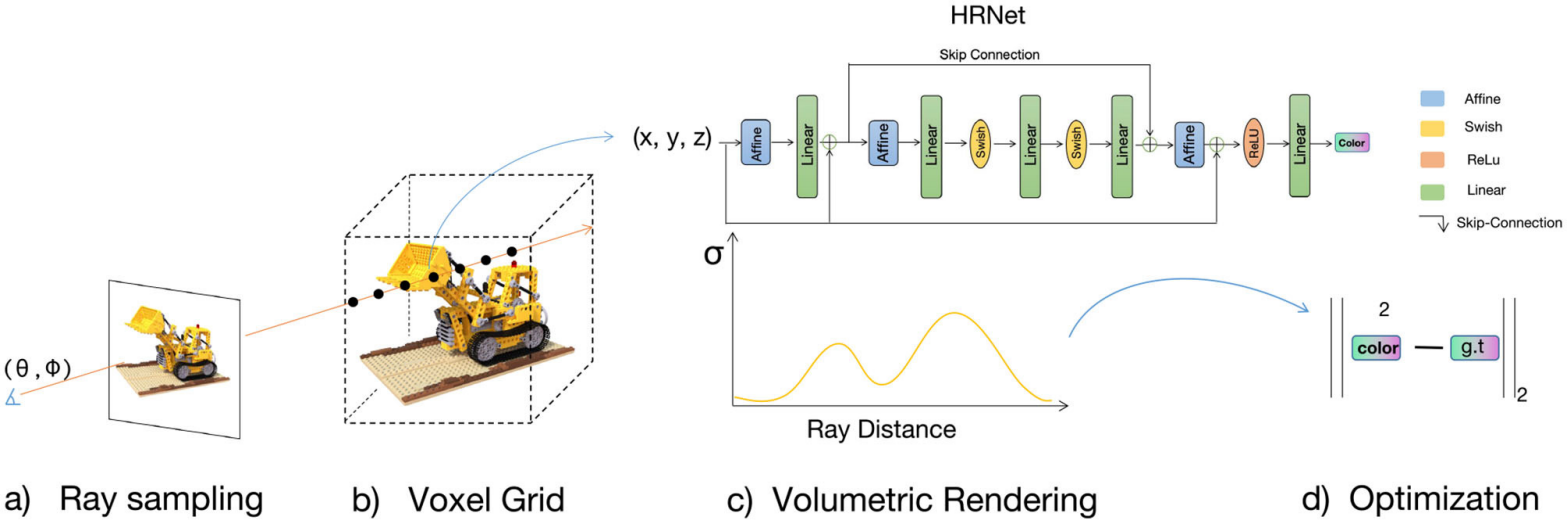
Method overview. The coordinates ***x*** of the ray sampling points are linearly interpolated by the learnable 3D Grid and then stitched with the viewpoint vector ***d***. Finally, they are fed into the HRNet to predict the color values. The HRNet is stably trained and accelerates convergence using an affine transformation layer. Layerscale and Skip Connection are used to enhance the network's ability to fit high-frequency information.

Although NeRF employs positional encoding that maps the inputs to a higher dimensional space using high-frequency functions to improve renderings that perform poorly at representing high-frequency variation in color and geometry, it still renders poorly on specular surfaces. Figure 1 shows that NeRF and its variants rendering quality is still not ideal on specular objects. The rendering results for the drums category appear notably rough. These rough artifacts are main caused by spectral bias (Rahaman et al., 2019). Variants (Müller et al., 2022; Rosu and Behnke, 2023; Garbin et al., 2021; Hedman et al., 2021; Lindell et al., 2021; Liu et al., 2020; Rebain et al., 2021; Schwarz et al., 2020; Yu et al., 2021) of NeRF work well in the direction of acceleration, but there is little work that combines acceleration with image quality, especially for scene highlight details. A deeper MLP with ReLU activation is used to implicitly represent the 3D scene. Additionally, position encoding (Rahaman et al., 2019) is applied to the sampled point coordinates and viewpoint vector, mapping them to a high-dimensional space and enhancing the MLP's ability to approximate high-frequency functions. This MLP+ReLU implicit representation structure has not been effectively improved in the subsequent NeRFs method, leading to a long-term limitation in the image rendering quality of NeRFs, where the model cannot effectively learn the high-frequency details of the image. To address this issue, we propose the HRNet architecture, which can be combined with a learnable 3D grid to significantly enhance image rendering quality and achieve satisfactory model training speed. The MLP architecture employed by NeRFs (Mildenhall et al., 2020) can lead to variations in their feature vectors with depth, inconsistencies in feature space, and issues such as gradient loss and explosion. Hence, we incorporate skip connections, layer scaling, and affine layers into the traditional MLP to ensure stable training and enhance the network's capacity to model high-frequency components. Simultaneously, we utilize the Swish activation function to replace ReLU. This alteration significantly enhances the MLP's capability to capture image details. Our method overview is shown in Figure 2.

To summarize, we make the following contributions:

1. We propose the Highlight Recovery Network (HRNet), an enhanced MLP that effectively renders scene image details, especially for highlight scenes.

2. We utilize explicit and discretized volumetric representations for modeling. While not a new approach, when combined with HRNet, it achieves leading rendering speed and image quality. Compared to NeRF, our method requires only about 18 min to train a single scene and improves the average PSNR by 3–5 dB.

## 2 Related work

### 2.1 Neural radiance fields

Recently, NeRF has caused a new boom in new view synthesis tasks. By simply inputting images of the sparse angles of the scene and the corresponding camera parameters, images of the new view can be obtained. Compared to traditional explicit and discrete volume representations such as voxel lattices and MPI, NeRF performs extremely well in the novel view synthesis task, using a coordinate-based MLP as an implicit and continuous volume representation. NeRF achieves appealing quality and has good flexibility with many follow-up extensions to various setups, e.g., relighting (Bi et al., 2020; Boss et al., 2021; Srinivasan et al., 2021; Zhang et al., 2021), deformation (Gafni et al., 2021; Noguchi et al., 2021; Park et al., 2020; Tretschk et al., 2021), self-calibration (Jeong et al., 2021; Lin et al., 2021; Yen-Chen et al., 2021; Meng et al., 2021; Wang et al., 2021), meta-learning (Tancik et al., 2021), dynamic scene modeling (Gao et al., 2021; Li et al., 2021; Martin-Brualla et al., 2021; Pumarola et al., 2021; Xian et al., 2021), and generative modeling (Chan et al., 2021; Kosiorek et al., 2021; Schwarz et al., 2020). However, NeRF and its variants require a lengthy training time ranging from hours to days for a single scene. Here, we introduce the derivation of the density voxel grid to accelerate the model.

### 2.2 Enhanced standard MLP

As a classic neural network, MLP is applied to various tasks of deep learning. Transformers (Vaswani et al., 2017) built by MLP shine in natural language processing, image classification, and recognition tasks. MLP-Mixer (Tolstikhin et al., 2021) uses Mixer's MLP to replace ViT's Transformer (Dosovitskiy et al., 2020), which reduces the degree of freedom of feature extraction and can cleverly exchange information between patches and information within patches alternately. Recently, Facebook AI Lab proposed ResMLP (Touvron et al., 2021) for tasks such as image classification, a purely MLP-based architecture that uses residual operations to update projection features, and finally average pools all block features classification later. It is more stable than Transformer training and more concise than MLP-Mixer. Inspired by ResMLP, we propose the HRNet architecture to represent neural radiance fields, which has amazing performance.

### 2.3 Spectral bias

Recent works (Mildenhall et al., 2020; Rahaman et al., 2019; Sitzmann et al., 2020; Tancik et al., 2020) have shown that a standard MLP with ReLU (Glorot et al., 2011) shows limited performance in representing high-frequency textures. Researchers call this phenomenon spectral bias. Its presence leads to some limitations of the coordinate-based MLP to implicitly represent 3D scenes, such as the inability to fit high-frequency details of object surfaces. Various methods have been proposed to alleviate this problem. For example, researchers have proposed the SIREN (Sitzmann et al., 2020) periodic activation function to replace the ReLU activation function, which can achieve accelerated convergence as well as improved image quality. Other approaches (Mildenhall et al., 2020; Tancik et al., 2020) are to map input coordinates into high-dimensional Fourier space by using position encoding or Fourier feature mapping before passing an MLP. This is also the scheme used by NeRFs, but we found that the images rendered by NeRFs still have problems with highlight details being difficult to capture. We consider that the ReLU activation function is still not the optimal choice, so we use the Swish (Ramachandran et al., 2017) activation function to replace ReLU, and the introduction of the skip-connection, layerscale and affine module in MLP can ensure the consistency of network features and significantly improve the network's ability to fit high-frequency details.

## 3 Preliminaries

To represent 3D scenes implicitly, NeRF (Mildenhall et al., 2020) employs MLP networks. Given any input 3D position ***x*** and a viewing direction ***d***, NeRF uses a spatial MLP to output the density ***σ*** of volumetric particles and view-dependent color emission ***c***:


(1a) $$\begin{array}{l} \begin{array}{c} \left(\sigma ,e\right)={\text{MLP}}^{\left(\text{post}\right)}\left(\gamma \left(x\right)\right) \end{array} \end{array}$$

(1b) $$c={\text{MLP}}^{\left(\text{rgb}\right)}(e,\gamma (d))$$

MLP^(post)^ first processes the input 3D coordinate × with eight fully-connected layers and outputs ***σ*** and a feature vector ***e***. This feature vector ***e*** is then concatenated viewing direction ***d*** and passed to MLP^(rgb)^ that output the view-dependent RGB color ***c***. In practice, positional encoding ***γ*** is applied to ***x*** and ***d***, which enables the MLPs to learn the high-frequency details from low-dimensional input (Tancik et al., 2020). The ray is projected at the pixel center of the image along the viewing direction ***d***, and ***N*** coordinate points are sampled in a limited range. The MLP is used to query their densities and colors of these ***N*** points. Finally, the ***N*** queried results are accumulated into a single color with the volume rendering quadrature in accordance with the optical model given by Max (1995):


(2a) $$\hat{C}(r)=\sum_{i=1}^{N}T_{i}\left(1-\exp\left(-\sigma_{i}\delta_{i}\right)\right)c_{i})$$

(2b) $$\begin{array}{l} \text{where}T_{i}=\exp\left(-\overset{i-1}{\underset{j=1}{\sum }}\sigma_{j}\delta_{j}\right) \end{array}$$

where δ_*j*_ is the distance between adjacent samples. The function *T*_*i*_ denotes accumulated transmittance along the ray from near and far samples. If the ray is blocked, the later sample points will not be calculated. For more accurate sampling, NeRF simultaneously optimize two networks: one “coarse” and one “fine.” Given a training image with camera pose, the NeRF model optimizes the loss value between the predicted pixel value and the true pixel value:


(3) $$\begin{array}{l} L=\underset{r\in R}{\sum }\left[||{Ĉ}_{c}\left(r\right)-C\left(r\right)|{|}_{2}^{2}+||{Ĉ}_{f}\left(r\right)-C\left(r\right)|{|}_{2}^{2}\right] \end{array}$$

where Ĉ_*c*_(***r***) and Ĉ_*f*_(***r***) is color pixel values for the coarse and fine network outputs. *C*(***r***) is the ground truth. R is the set of rays in each batch.

## 4 Method

**Figure 3:**
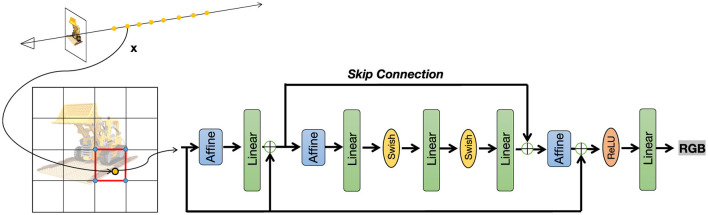
HRNet overview. The proposed High-Resolution Residual Multi-Layer Perceptron Network comprises Affine transformations, linear layers, Swish activation, and residual connections, enabling efficient feature learning.

In this section, similar to Sun et al. (2022), we will first introduce how to use a density voxel grid to achieve scene reconstruction. The reconstruction process is divided into two stages: a coarse stage and a fine stage. In the coarse stage, a low resolution voxel grid is used to obtain the density and color of the scene through an interpolation algorithm. Building on the coarse stage, the grid resolution is then increased to further reconstruct scene details and view-dependent colors. Next, we propose HRNet, a novel High-Resolution Residual Multi-Layer Perceptron (MLP) Network designed to effectively model high-dimensional data through a hierarchical residual architecture. HRNet leverages a combination of affine transformations, multi-layer perceptrons, and a custom Swish activation function to achieve robust feature extraction and transformation, culminating in a low-dimensional output suitable for tasks such as 3D regression or scene representation. Figure 3 illustrates the overall network structure of our method.

### 4.1 Coarse scene reconstruction

In the coarse stage, our method aims to efficiently establish an initial representation of the geometry and density distribution of the scene. This stage employs a low-resolution voxel grid to accelerate the optimization process, prioritizing computational efficiency over fine-grained detail. The primary objective is to provide a robust foundation for subsequent refinement by capturing the overall structure and appearance of the scene. A voxel grid $V_{\text{density}}^{\left(c\right)}\in {\mathbb{R}}^{N^{\left(c\right)}\times N^{\left(c\right)}\times N^{\left(c\right)}}$ is used to explicitly model the volumetric density of the scene, where *N*^(*c*)^ denotes the resolution of the coarse grid (e.g. *N*^(*c*)^ = 64). For any 3D position *x*∈ℝ^3^, the density σ(*x*) is computed via trilinear interpolation:


$$\sigma \left(x\right)=\text{Interpolate}\left(V_{\text{density}}^{\left(c\right)},x\right)$$

Similarly, a separate voxel grid $V_{\text{rgb}}^{\left(c\right)}\in {\mathbb{R}}^{3\times N^{\left(c\right)}\times N^{\left(c\right)}\times N^{\left(c\right)}}$ stores view-invariant color emissions. The color *c*(*x*) at position *x* is obtained as:


$$c\left(x\right)=\text{Interpolate}\left(V_{\text{rgb}}^{\left(c\right)},x\right)$$

This stage effectively captures the coarse geometry and appearance, serving as an initialization for the fine stage while avoiding the computational overhead of high-resolution optimization from scratch.

### 4.2 Fine scene reconstruction

Building upon the coarse stage, the fine stage refines the representation of the scene by improving geometric details and introducing view-dependent appearance effects. The resolution of the voxel grid is increased to *N*^(*f*)^ (e.g. *N*^(*f*)^ = 256), enabling the method to model intricate structures and subtle variations in density and color with greater precision. The density is now represented by a higher resolution voxel grid $V_{\text{density}}^{\left(f\right)}\in {\mathbb{R}}^{N^{\left(f\right)}\times N^{\left(f\right)}\times N^{\left(f\right)}}$, and the density at position *x* is similarly interpolated:


$$\sigma \left(x\right)=\text{Interpolate}\left(V_{\text{density}}^{\left(f\right)},x\right)$$

To account for view-dependent effects, we introduce a feature voxel grid $V_{\text{feature}}^{\left(f\right)}\in {\mathbb{R}}^{F\times N^{\left(f\right)}\times N^{\left(f\right)}\times N^{\left(f\right)}}$, where *F* denotes the dimensionality of the feature (e.g. *F* = 16). The color *c*(*x, d*) is computed by combining the interpolated features with the viewing direction *d* through HRNet:


$$c\left(x,d\right)=\text{HRNet}\left(\text{Interpolate}\left(V_{\text{feature}}^{\left(f\right)},x\right),d\right)$$

The HRNet maps the input features and direction to an RGB output. The output of the coarse stage is frozen or used as a priori to guide the optimization of $V_{\text{density}}^{\left(f\right)}$ and $V_{\text{feature}}^{\left(f\right)}$, thus reducing the convergence time. This two-stage approach balances efficiency and quality, achieving superior rendering precision while maintaining computational tractability.

### 4.3 HRNet: high-resolution residual multi-layer perceptron network

#### 4.3.1 Swish activation function

HRNet incorporates a parameterized Swish activation function, defined as *f*(*x*) = *x*·σ(*x*), where σ(*x*) denotes the sigmoid function. This activation is implemented with an optional in-place operation to optimize memory usage during training. Unlike the widely-used ReLU, Swish provides a smooth, non-monotonic nonlinearity that preserves negative values, potentially enhancing gradient flow and model expressiveness.

#### 4.3.2 Affine transformation module

A lightweight Affine module is introduced to perform per-dimension scaling and shifting of the input features. Formally, given an input *x*∈ℝ^*d*^, the transformation is computed as *y* = α·*x*+β, where α, β∈ℝ^*d*^ are learnable parameters initialized to ones and zeros, respectively. This module serves as a feature normalization mechanism, akin to simplified layer normalization, enabling the network to adaptively adjust the scale and bias of intermediate representations.

#### 4.3.3 Multi-layer perceptron (MLP) block

The MLP block in HRNet consists of a three-layer fully-connected network with an expansion-compression design. For an input dimension *d*, the architecture expands the feature space to 2*d* through the first layer, maintains this dimensionality in the second layer, and compresses it back to *d* in the third layer. Each linear transformation is followed by the Swish activation, facilitating non-linear feature mapping. This bottleneck-inspired design increases the network's capacity to capture complex patterns while maintaining computational efficiency.

#### 4.3.4 Residual MLP block (ResMLP block)

At the core of HRNet lies the Residual MLP (ResMLP) Block, which integrates residual connections and a LayerScale mechanism to enhance training stability and performance in deep architectures. The block operates as follows:

- The input *x* is first processed by an Affine module, followed by a linear layer Linear(*d, d*), yielding a residual term *r*_1_.

- This residual is added to the input, and further scaled by a learnable LayerScale parameter $\lambda_{1}\in {\mathbb{R}}^{d}$, initialized with a small constant (e.g., 10^−5^).

- The resulting feature is then passed through another Affine module and the MLP block, producing a second residual term *r*_2_, which is similarly scaled by $\lambda_{2}\in {\mathbb{R}}^{d}$ and added to the intermediate representation.

This dual-residual structure, combined with LayerScale, mitigates the vanishing gradient problem and allows the network to preserve high-resolution feature information across layers.

#### 4.3.5 HRNet architecture

The full HRNet model consists of a stack of *N* ResMLP blocks, where *N* is a configurable depth parameter (we set N = 1 in our experiments). The input *x* is sequentially processed by each block, followed by a final Affine transformation and a global residual connection that adds the original input to the output of the block stack. The resulting features are activated using ReLU and mapped to a 3-dimensional output via a linear layer Linear(*d*, 3). This design ensures that both local and global contextual information are preserved, making HRNet particularly suited for tasks requiring precise low-dimensional predictions.

#### 4.3.6 Design rationale and contributions

HRNet draws inspiration from residual networks and Transformer-like architectures, adapting these concepts to a fully-connected MLP framework. The use of LayerScale and multiple residual paths enables the model to scale effectively with depth, while the Swish activation and affine transformations enhance its representational flexibility. By outputting a 3-dimensional vector, HRNet is tailored for applications in 3D vision tasks, such as scene reconstruction or object localization, where maintaining high-resolution feature fidelity is critical.

## 5 Experiments

In this section, we highlight the advantages of our method by comparing experimental data and rendered images. We use four publicly available datasets for this comparison: Synthetic-NeRF (Mildenhall et al., 2020), Synthetic-NSVF (Liu et al., 2020), BlendedMVS (Yao et al., 2020), and Tanks&Temples (Knapitsch et al., 2017). We will first introduce each dataset, followed by images rendered using our method. Next, we present the comparison results using standard metrics from previous view synthesis studies: PSNR, SSIM (Wang et al., 2004), and LPIPS (Zhang et al., 2018). Note that golden yellow indicates first place, orange indicates second, and light yellow indicates third.

### 5.1 Synthetic scenes

#### 5.1.1 Synthetic-NeRF

**Figure 4:**
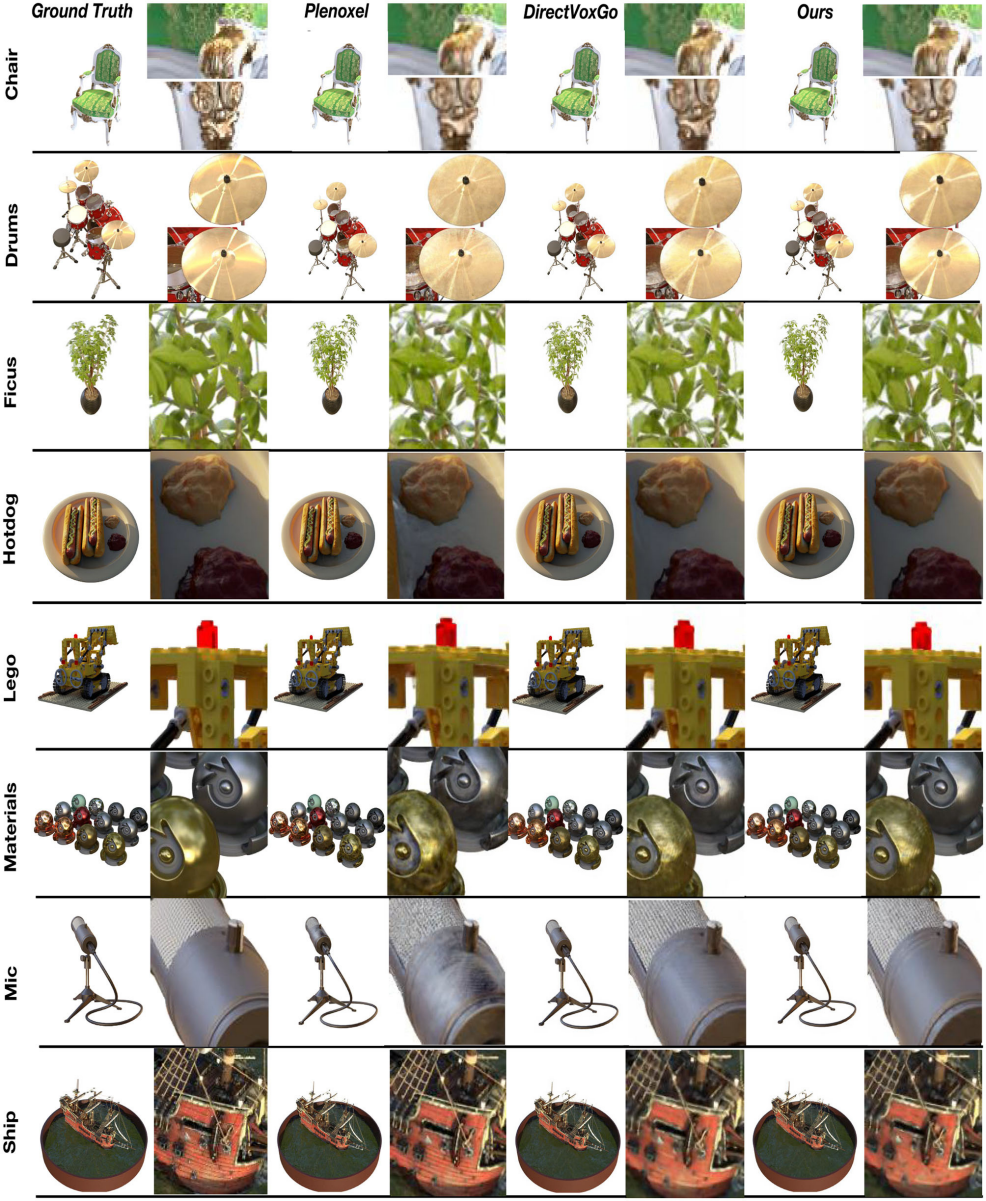
Quantitative results on the synthetic-NeRF test scenes. We selected three categories of rendered images from Synthetic-NeRF: Hotdog, Mic, and Materials to compare with Plenoxels and DirectVoxGo. We can find that the images rendered by Plenoxels and DirectVoxGo are not smooth enough, and the blue box dotted area in the figure.

The Synthetic-NeRF dataset, from the original NeRF paper, features path-traced renderings of geometrically complex objects with non-Lambertian materials across eight scenes (chair, drums, ficus, hotdog, lego, materials, mic, ship). Each scene includes 100 training, 100 validation, and 200 test images at 800 × 800 resolution in RGBA format. We conducted comprehensive experiments on this public dataset, comparing 13 leading methods, including DirectVoxGo, Plenoxels, PlenOctrees, Mip-NeRF, and NSVF, for image rendering quality. Table 1 highlights our method's superior performance in PSNR, SSIM, and LPIPS metrics. While Mip-NeRF excels in quality, it requires 6 h to train on an RTX 2080Ti GPU. Figure 4 compares our method against state-of-the-art approaches, showing zoomed-in renderings from eight scenes. Our method produces sharper, more detailed images with fewer artifacts, closely resembling ground truth.

**Table 1:** Quantitative results on the Synthetic-NeRF test scenes.

**Methods**	**PSNR↑**	**SSIM↑**	**LPIPS↓**
SRN (Sitzmann et al., 2019)	22.26	0.846	0.170
NV (Lombardi et al., 2019)	26.05	0.893	0.160
NeRF (Mildenhall et al., 2020)	31.01	0.947	0.081
JaxNeRF (Deng et al., 2020)	31.69	0.953	0.068
Mip-NeRF (Barron et al., 2021)	33.09	0.961	0.043
AutoInt (Lindell et al., 2021)	25.55	0.911	–
FastNeRF (Garbin et al., 2021)	29.90	0.937	–
SNeRG (Hedman et al., 2021)	30.38	0.950	–
NSVF (Liu et al., 2020)	31.74	0.953	–
PlenOctrees (Yu et al., 2021)	31.71	0.958	0.053
Plenoxels (Fridovich-Keil et al., 2022)	31.71	0.958	0.049
DirectVoxGo (Sun et al., 2022)	31.93	0.956	0.053
KiloNeRF (Reiser et al., 2021)	31.00	0.950	–
Ours	32.50	0.960	0.048

#### 5.1.2 Synthetic-NSVF

The Synthetic-NSVF dataset includes eight diverse objects (Wineholder, Steamtrain, Toad, Robot, Bike, Palace, Spaceship, Lifestyle), each with 100 training and 200 test images at 800 × 800 resolution. Its complex geometries and textures challenge rendering methods. As shown in Table 2, our method ranks first in PSNR (35.83) and LPIPS (0.015), nearly matching NSVF's top SSIM (0.979 vs. 0.978), and outperforms DirectVoxGo by 0.8 dB in PSNR. This demonstrates our approach's superior quality, fidelity, and artifact reduction in synthetic scene rendering.

**Table 2:** Quantitative results on the Synthetic-NSVF test scenes.

**Methods**	**PSNR↑**	**SSIM↑**	**LPIPS↓**
SRN (Sitzmann et al., 2019)	24.33	0.882	0.141
NV (Lombardi et al., 2019)	25.83	0.892	0.125
NeRF (Mildenhall et al., 2020)	30.81	0.952	0.043
NSVF (Liu et al., 2020)	35.13	0.979	0.015
DirectVoxGo (Sun et al., 2022)	35.08	0.975	0.019
KiloNeRF (Reiser et al., 2021)	33.37	0.970	–
Ours	35.83	0.978	0.015

#### 5.1.3 BlendedMVS and Tanks & Temples

These datasets, characterized by real-world complexity such as intricate textures, varied lighting, and complex geometries, are established benchmarks for assessing image rendering in photogrammetry and 3D reconstruction. Our method outperforms on BlendedMVS and Tanks & Temples datasets (Tables 3, 4), improving PSNR by 4 dB and 3 dB, respectively, against NeRF baselines using public real-world data. This reflects our approach's strength in capturing fine details and handling diverse, challenging scenes—including reflective surfaces, shadows, and occlusions—enhancing rendering fidelity across varied datasets.

**Table 3:** Quantitative results on the BlendedMVS test scenes.

**Methods**	**PSNR↑**	**SSIM↑**	**LPIPS↓**
SRN (Sitzmann et al., 2019)	20.51	0.770	0.294
NV (Lombardi et al., 2019)	23.03	0.793	0.243
NeRF (Mildenhall et al., 2020)	24.15	0.828	0.192
NSVF (Liu et al., 2020)	26.90	0.898	0.113
DirectVoxGo (Sun et al., 2022)	28.02	0.922	0.075
KiloNeRF (Reiser et al., 2021)	27.39	0.920	–
Ours	28.50	0.929	0.069

**Table 4:** Quantitative results on the Tanks&Temples test scenes.

**Methods**	**PSNR↑**	**SSIM↑**	**LPIPS↓**
SRN (Sitzmann et al., 2019)	24.09	0.847	0.251
NV (Lombardi et al., 2019)	23.70	0.834	0.260
NeRF (Mildenhall et al., 2020)	25.78	0.864	0.198
JaxNeRF (Deng et al., 2020)	27.94	0.904	–
NSVF (Liu et al., 2020)	28.40	0.900	0.153
PlenOctrees (Yu et al., 2021)	27.99	0.917	–
DirectVoxGo (Sun et al., 2022)	28.41	0.911	0.148
KiloNeRF (Reiser et al., 2021)	28.41	0.910	–
Ours	28.82	0.920	0.137

### 5.2 Ablation study

We study the impact of the different components of HRNet in ablation studies. We mainly validate the effectiveness of layerscale, skip-connection and swish —which enable standard MLP to model scene appearance with NeRF better quality. At the same time, verify the validity of the case without position encoding. Table 5 shows that our HRNet still renders images of high quality even without positional encoding. When we remove the some module, the evaluation metrics drop significantly, which fully demonstrates the effectiveness of this module.

**Table 5:** Ablation studies.

**Methods**	**PSNR↑**	**SSIM↑**	**LPIPS↓(Vgg)**
NeRF (Mildenhall et al., 2020)	31.01	0.947	0.081
Ours	32.52	0.959	0.048
Ours (no pe)	32.50	0.959	0.049
Ours (no layerscale)	32.16	0.958	0.050

## 6 Conclusion

Our method strikes a balance between rendering quality and speed in neural radiance fields, surpassing both the original NeRF and most of its variants in terms of rendering quality and training efficiency. As noted in the introduction, our approach trains a single scene in ~18 min, with a PSNR improvement of 3–5 dB over the original NeRF. However, it still falls short of achieving real-time rendering and shows some flaws in quality at higher resolutions. Despite these limitations, we believe our method lays the groundwork for faster convergence and enhanced rendering quality in such scenarios. We expect that our approach will contribute to further advancements in NeRF-based scene reconstruction and its applications.

## Data Availability

The original contributions presented in the study are included in the article/supplementary material, further inquiries can be directed to the corresponding author.
